# Astragaloside IV attenuates high glucose-induced EMT by inhibiting the TGF-β/Smad pathway in renal proximal tubular epithelial cells

**DOI:** 10.1042/BSR20190987

**Published:** 2020-06-23

**Authors:** Ya-Ning Wang, Shi-Li Zhao, Yan-Yan Su, Jun-Xia Feng, Shuai Wang, Xiao-Ming Liao, Li-Na Wang, Jing-Chun Li, Ping Meng, Hong-Yan Li, Yun-Fang Zhang

**Affiliations:** 1 Department of Nephrology, Binzhou Medical University Hospital, Binzhou 256603, China; 2 Department of Nephrology, Affiliated Huadu Hospital, Southern Medical University (People's Hospital of Huadu District), Guangzhou 510800, China; 3 The Third School of Clinical Medicine, Southern Medical University, Guangzhou 510800, China; 4 Department of Critical Care Medicine, Guangzhou Hospital of Integrated Traditional and West Medicine, Guangzhou 510800, China; 5 Department of The Combination of Chinese and Western Medicine, Huadu District People’s Hospital, Southern Medical University, Guangzhou 510800, China

**Keywords:** Astragalosides IV, epithelial-to-mesenchymal transition, TGF-β/Smad pathway, tubular epithelial cells

## Abstract

In the present study, we examined the molecular mechanism of astragaloside IV (AS-IV) in high glucose (HG)-induced epithelial-to-mesenchymal transition (EMT) in renal proximal tubular epithelial cells (PTCs). NRK-52E cell viability and apoptosis were determined by the cell counting kit-8 (CCK-8) assay and flow cytometric analysis, respectively. Expressions of E-cadherin, N-cadherin, vimentin, and occludin were measured by Western blot, and those of E-cadherin and N-cadherin were additionally measured by immunofluorescence analysis. Transforming growth factor-β1 (TGF-β1) and α-smooth muscle actin (α-SMA) expressions were detected by quantitative real-time polymerase chain reaction (qRT-PCR) and Western blot. The expressions of Smad2, Smad3, phosphorylated-Smad2 (p-Smad2), and p-Smad3 were measured using Western blot. We found that AS-IV could recover NRK-52E cell viability and inhibit HG-induced cell apoptosis. TGF-β1, α-SMA, Smad2, Smad3, p-Smad2, and p-Smad3 expressions were decreased in the AS-IV-treated groups compared with the HG group. Moreover, the expressions of E-cadherin and occludin were remarkably up-regulated and those of N-cadherin and vimentin were down-regulated in the AS-IV-treated groups compared with the HG group. Interestingly, the TGF-β1 activator SRI-011381 hydrochloride had an antagonistic effect to AS-IV on HG-induced EMT behavior. In conclusion, AS-IV attenuates HG-induced EMT by inhibiting the TGF-β/Smad pathway in renal PTCs.

## Introduction

Diabetic kidney disease (DKD) is a common complication of diabetic microvascular lesions of the kidneys caused by glomerular sclerosis, often leading to end-stage renal disease (ESRD), a frequent cause of death in patients with diabetes [1–3]. Irrespective of the etiology, tubulointerstitial fibrosis (TIF) is the final and frequent manifestation of ESRD [4]. Factors contributing to ESRD include pro-inflammatory factor production, inflammatory cell infiltration, fibroblast proliferation and activation, increased extracellular matrix (ECM), and tubular epithelial-to-mesenchymal cell transformation (EMT). Recent studies have demonstrated that high glucose (HG)-induced tubular EMT is a central event in mediating TIF [5–8]. Therefore, understanding the pathogenesis of diabetic nephropathy (DN) and the mechanism of EMT is essential to improve prognosis in patients with DKD.

The process of tubular EMT involves the following four steps: (1) perdition of epithelial cell adhesion; (2) expression of *de novo* α-smooth muscle actin (α-SMA) and reorganization of actin; (3) disruption of the tubular basement membrane; and (4) elevation of cell migration and invasion [9,10]. During EMT, mesenchymal markers such as α-SMA and vimentin are induced and epithelial cell markers such as E-cadherin, which are essential for the structural integrity of the renal epithelium, are eliminated [11,12]. In addition, EMT has been reported to require the participation of growth factors or cytokines and integration of multiple signal pathways at different stages [9,11,13,14]. Of these factors, transforming growth factor-β (TGF-β) is a principal mediator of EMT that functions by activating the Smad signaling pathway [13,14]. Although the molecular regulation of EMT has been extensively studied in renal cells, little is known about the mechanisms linking EMT to cellular transport dysfunction in renal proximal tubular epithelial cells (PTCs). Therefore, it is important to explore the mechanism of EMT to establish novel effective therapeutic strategies that can prevent or arrest progressive renal failure.

Astragaloside IV (AS-IV) is a key active component of the medicinal herb *Astragalus membranaceus*, which is widely used to treat several diseases including cardiovascular and digestive disorders [15,16]. After AS-IV was administered in rats, pharmacokinetic analysis revealed that the drug was highly accumulated in the liver and kidneys, followed by the heart and lungs [1], implying its plausible protective role in the liver and kidneys. AS-IV inhibited neutrophil aggregation and protected nerve cells by inhibiting the NF-κB pathway [17]. AS-IV inhibited liver fibrosis induced by porcine serum, possibly by reducing collagen synthesis, decreasing liver TGF-β1 expression, and inhibiting excessive proliferation of stellate cells [18]. Recently, increasing number of studies conducted using various kidney injury models have indicated the specific protective effect of AS-IV [19,20]. Gui et al. demonstrated that AS-IV could improve renal ischemia–reperfusion and cisplatin-induced acute kidney injury by reducing oxidative stress and apoptosis [19]. Moreover, Qi et al. reported that AS-IV intervention improved TIF occurrence by particularly reducing oxidative stresses in renal PTCs [20]. Che et al. and Fujimoto et al. demonstrated that AS-IV inhibited HG-induced damage to renal PTCs by promoting expressions of hepatocyte growth factor, its receptor (c-met) system, and p38 MAPK in the TGF-β1 signaling pathway [8,21]. AS-IV appears to delay TGF-β1-, Smad2-/3-, and α-SMA-induced renal fibrosis in diabetic mice [22]. These results support the important role of AS-IV in renal TIF. However, it is unclear how AS-IV affects EMT in renal PTCs. In the present study, we aimed to investigate the action mechanism of AS-IV on EMT in renal PTCs, which could help design effective strategies to treat DKD.

## Materials and methods

### Cell culture and treatment

The normal rat kidney tubular epithelial cell line NRK-52E was obtained from ATCC (Manassas, VA, U.S.A.), and the cells were cultured in Dulbecco’s modified Eagle’s medium (Gibco-BRL, Gaithersburg, MD) supplemented with 10% fetal bovine serum (FBS) at 37°C in a humidified atmosphere of 5% CO_2_. After reaching 70–80% confluence, the cells were subjected to serum deprivation before experimental manipulations. To determine the effect of AS-IV on HG-stimulated NRK-52E cells, different concentrations of AS-IV (20, 40, 80, and 100 μg/ml) and the TGF-β1 activator SRI-011381 hydrochloride (10 μM) were added to the media 24 h after treatment with HG. Meanwhile, we also added equivalent volume of DMSO as negative control (NC) group. For the TGF-β1 activator treatment, SRI-011381 hydrochloride (10 μM) was added to the media at 4 h after AS-IV treatment. NRK-52E cells without HG served as the control group. For HG stimulation, 30 mM of glucose was chosen, according to a previous report [8].

### Cell viability analysis

NRK-52E cell viability was determined using the cell counting kit-8 (CCK-8, CK04, Dojindo) assay. Cells were seeded on to 96-well plates at a concentration of 10^5^/ml and allowed to adhere in FBS-free media before being submitted to cytometry. After adherence, the cells were stimulated with different concentrations (0–100 μg/ml) of AS-IV for 24 h, after which 10 μl of CCK-8 was added to each well and incubated at 37°C for 1 h. The absorbance of CCK-8 was read at 450 nm using a microplate reader. Cell viability was expressed as the surviving fraction.

### Cell apoptosis by flow cytometric analysis

After treatment, NRK-52E cells from different groups were harvested and rinsed with phosphate buffered saline (PBS). The apoptotic rate was detected using the Annexin V-FITC/PI apoptosis kit (40302ES20, YEASEN, Shanghai), according to the manufacturer’s instructions.

### RNA extraction and quantitative real-time polymerase chain reaction

Total RNA was isolated from NRK-52E cells by using the TRIzol reagent (9109, Takara), according to the manufacturer’s instructions. Subsequently, total RNA was inversely transcribed into cDNA by using the Bestar™ quantitative real-time polymerase chain reaction (RT-qPCR) kit (2220, DBI, Denmark). To detect TGF-β1 and α-SMA expression levels, qRT-PCR was performed using an ABI PRISM 7500 Sequence Detection System (Life Technologies, Grand Island, NY, U.S.A.) with Bestar™ qPCR Master Mix (2043, DBI, Denmark). Reactions were performed in triplicate and contained 100 ng of cDNA, 0.5 μl of each primer (10 μm/μl), and 10 μl of the SYBR Green Master Mix in a final volume of 20 μl. The amplification reactions were performed under the following conditions: 95°C for 2 min, followed by 40 cycles of 94°C for 20 s, 58°C for 20 s, and 72°C for 20 s. The melting curve analysis was performed by increasing the temperature from 65 to 94°C (0.5°C per 10 s). The relative expression level for each sample was calculated using the 2^−△△*C*_t_^ method, as was described by Livak and Schmittgen [23]. The following primer sequences, synthesized by RiboBio (Guangzhou, China), were used: GAPDH sense: 5′-CCTCGTCTCATAGACAAGATGGT-3′, GAPDH antisense: 5′-GGGTAGAGTCATACTGGAACATG-3′, α-SMA sense: 5′-CATCACCAACTGGGACGACA-3′, α-SMA antisense: 5′-TCCGTTAGCAAGGTCGGATG-3′, TGF-β1 sense: 5′-GACTCTCCACCTGCAAGACC-3′, and TGF-β1 antisense: 5′-GGACTGGCGAGCCTTAGTTT-3′. *GAPDH* was used as the reference gene.

### Western blot analysis

Total protein was extracted from NRK-52E cells by using the RIPA lysis buffer (Beyotime, Shanghai, China), and the protein concentration was determined using the BCA protein assay kit (Thermo, U.S.A.). Equal amounts of proteins from lysates were loaded and separated using 10% sodium dodecyl sulfate/polyacrylamide gel electrophoresis and transferred on to polyvinylidene fluoride membranes (Millipore, MA, U.S.A.). After blocking with 5% non-fat milk for 1 h at room temperature, the membranes were incubated with primary antibodies for N-cadherin (1:1000, 14215, CST), E-cadherin (1:1000, 14472, CST), vimentin (1:400, ab8978, Abcam), occludin (1:1000, ab31721, Abcam), α-SMA (1:1000, ab32575, Abcam), Smad2 (1:2000, ab33875, Abcam), phosphorylated Smad2 (p-Smad2; 1:1500, ab53100, Abcam), Smad3 (1:4000, ab40854, Abcam), p-Smad3 (1:1000, ab52903, Abcam), TGF-β1 (1:400, ab92486, Abcam), and GAPDH (1:10000, ab8245, Abcam) at 4°C overnight. GAPDH was considered as the loading control. After washing with Tris-buffered saline with Tween, the membranes were incubated with horseradish peroxidase-conjugated secondary antibodies (1:400, BA1062, BOSTE) for 2 h at room temperature. The protein signal was detected and visualized using the ECL™ Western blot detection kit (Thermo Fisher Scientific, Rockford, IL) and the net optical density value of the bands was analyzed using the Image-Pro Plus 6.0 software.

### Immunofluorescence analysis

For immunofluorescence analysis, the cells were fixed using 4% paraformaldehyde for 30 min. Next, the cells were treated with Triton X-100 for 20 min after washing three times with PBS. The cells were then incubated with N-cadherin (1:200, 14215, CST) or E-cadherin (1:50, 14472, CST) antibody at 4°C overnight. Subsequently, the cells were rinsed several times and incubated with a secondary antibody (ab150115, Abcam) for 2 h. Cells were then pre-incubated with normal donkey serum for 1 h, followed by incubation with 4′,6-diamidino-2-phenylindole for 10 min. Finally, the cells were examined using a fluorescence microscope (LIONHEART LX, BioTek, VT, U.S.A.), with fluorescein isothiocyanate (488 nm) and Texas-Red (568 nm) as the excitation filters.

### Statistical analysis

The results are presented as mean ± standard deviation (SD). All statistical analyses were performed using the SPSS 17.0 software. The statistical significance for multigroup comparisons was assessed using the one-way ANOVA test, whereas that between the two groups was assessed using the Student’s *t* test. A *P*-value of <0.05 was considered statistically significant.

## Results

### AS-IV improves NRK-52E cell viability and inhibits cell apoptosis

**Figure 1:**
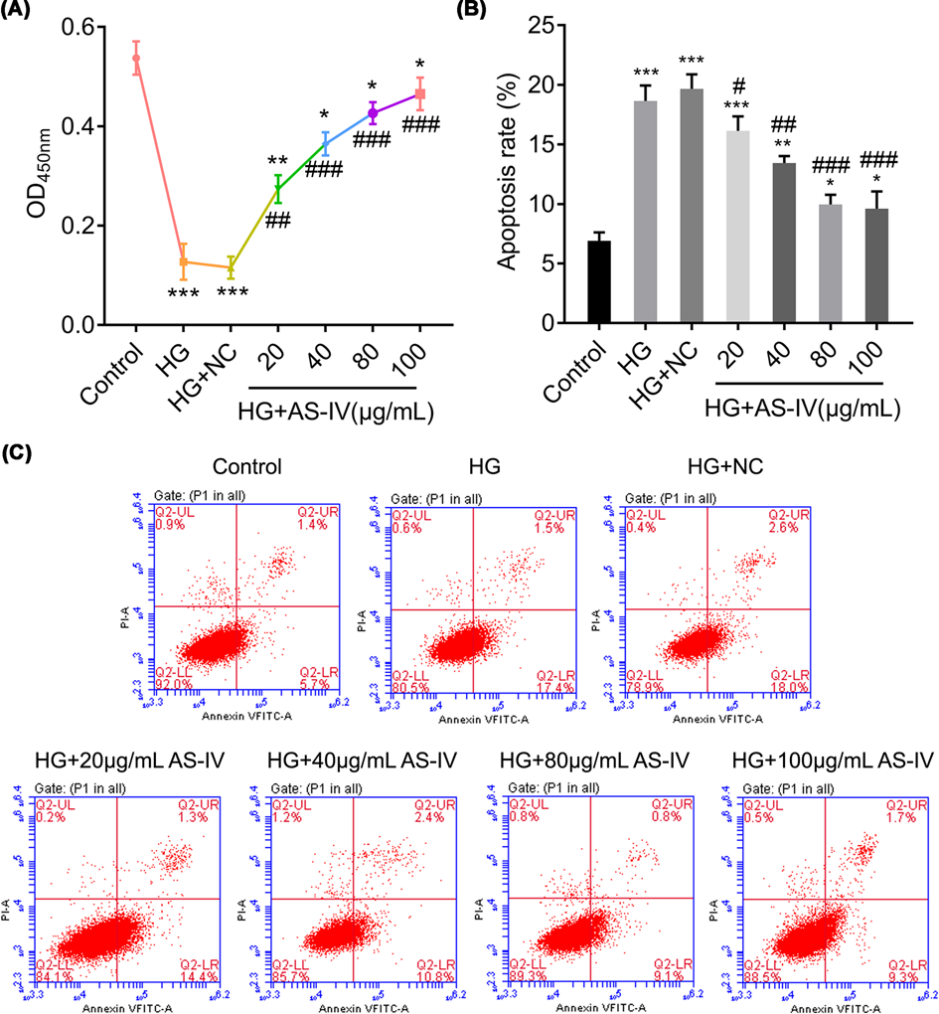
AS-IV modifies proliferation and apoptosis of NRK-52E cells (**A**) Proliferation rates of NRK-52E cells treated with AS-IV after HG-induced injury. (**B**) The apoptotic rate of HG-induced NRK-52E cells treated with AS-IV was evaluated and measured using flow cytometry (**C**). HG + NC, HG-induced NRK-52E cells with NC; HG + 20, 40, 80, or 100 μg/ml AS-IV, HG-induced NRK-52E cells treated with 20, 40, 80, or 100 μg/ml AS-IV treatment. NRK-52E cells without any treatment served as the control group. All data are presented as mean ± SD. *P<0.05, **P<0.01, ***P<0.001 compared with the control group. ^#^*P*<0.05, ^##^*P*<0.01, ^###^*P*<0.001 compared with the HG group.

To evaluate the role of AS-IV in the rat renal PTCs, NRK-52E cells were treated with HG for 24 h. Meanwhile, different concentrations of AS-IV were added to the medium and seven groups were established: HG, HG with NC, HG with 20 μg/ml of AS-IV, HG with 40 μg/ml of AS-IV, HG with 80 μg/ml of AS-IV, HG with 100 μg/ml of AS-IV, and non-treated NRK-52E cells, which served as the control group. As is shown in Figure 1A, the cell viability decreased significantly in the HG and HG with NC groups. However, cell viability improved in the HG with AS-IV groups in a dose-dependent manner. Moreover, HG treatment significantly enhanced cell apoptosis as compared with the control. However, the AS-IV-treated groups demonstrated enhanced cell apoptosis as compared with the HG and HG with NC groups, except the HG with 20 μg/ml of AS-IV group (Figure 1B,C). These results demonstrate the important role of AS-IV in NRK-52E cell viability and apoptosis.

### AS-IV reduces the TGF-β1 and Smad signaling pathway activation

**Figure 2:**
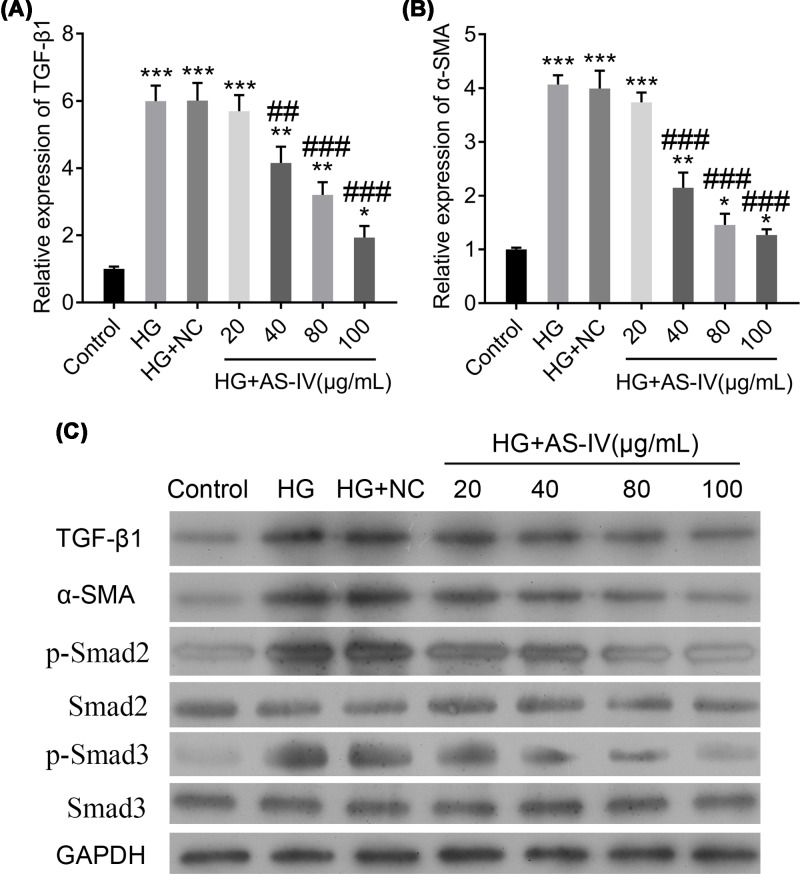
Changes in the expression of factors involved in the TGF-β1/Smad signaling pathway in different groups of NRK-52E cells (**A,B**) The relative expressions of TGF-β1 and α-SMA, respectively. (**C**) The protein changes of factors involved in the TGF-β1/Smad signaling pathway. Groups: HG; HG-induced NRK-52E cells; HG + NC, HG-induced NRK-52E cells with NC; HG + 20, 40, 80, or 100 μg/ml AS-IV; HG-induced NRK-52E cells treated with 20, 40, 80, or 100 μg/ml AS-IV. NRK-52E cells without any treatment served as the control group. All data are presented as mean ± SD. *P<0.05, **P<0.01, ***P<0.001 compared with the control group. ^##^*P*<0.01, ^###^*P*<0.001 compared with the HG group.

To investigate whether AS-IV is involved in the TGF-β1 and Smad signaling pathway, we measured the mRNA expressions of TGF-β1 and α-SMA. Their expressions were significantly increased in the HG and HG with NC groups, whereas they were significantly decreased in the AS-IV-treated groups, except the HG with 20 μg/ml of AS-IV group (Figure 2A,B). These results were further confirmed using the Western blot analysis (Figure 2C). Further analysis revealed that although the total protein concentrations of Smad2 and Smad3 were not changed in different groups, those of pSmad2 and pSmad3 were significantly enhanced in the HG and HG with NC groups as compared with the control group. However, the protein levels of p-Smad2 and p-Smad3 were significantly decreased in the AS-IV-treated groups, except in the HG with 20 μg/ml of AS-IV group (Figure 2C). These results imply the ability of AS-IV to reduce the activation of the TGF-β1 and Smad signaling pathway.

### AS-IV regulates epithelial cell marker and mesenchymal cell marker proteins

**Figure 3:**
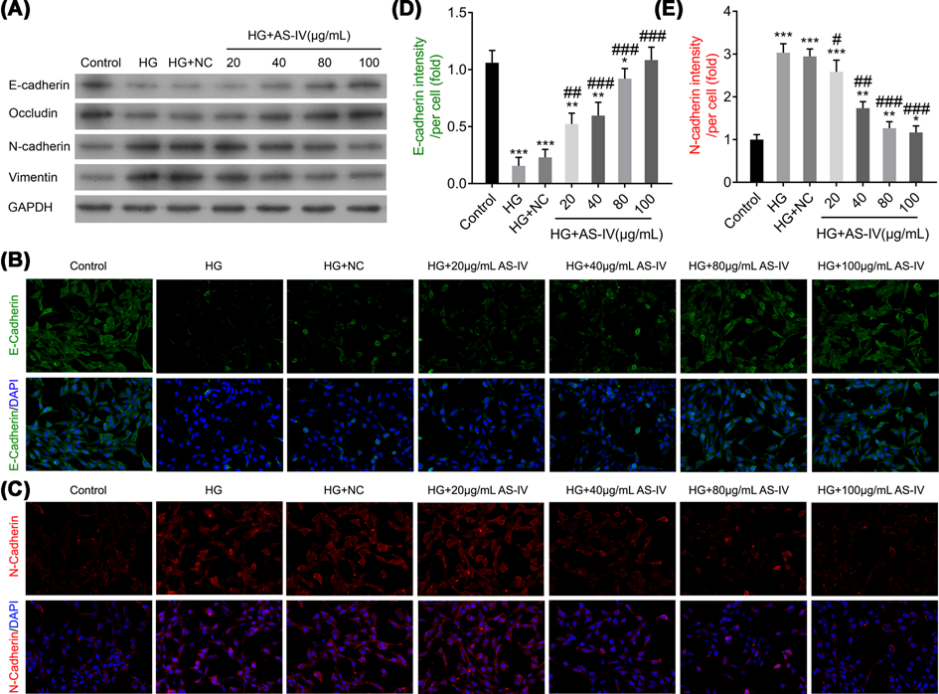
The effects of AS-IV on the expressions of E-cadherin, N-cadherin, vimentin, and occludin (**A**) The protein expression levels of E-cadherin, N-cadherin, vimentin, and occludin. (**B,C**) The protein expression levels of E-cadherin and N-cadherin as measured by the immunofluorescence assay. (**D,E**) The intensity per cell of E-cadherin and N-cadherin, respectively. Groups: HG; HG-induced NRK-52E cells; HG + NC, HG-induced NRK-52E cells with NC; HG + 20, 40, 80, or 100 μg/ml AS-IV; HG-induced NRK-52E cells treated with 20, 40, 80, or 100 μg/ml AS-IV. NRK-52E cells without any treatment served as the control group. All data are presented as mean ± SD. *P<0.05, **P<0.01, ***P<0.001 compared with the control group. ^#^*P*<0.05, ^##^*P*<0.01, ^###^*P*<0.001 compared with the HG group.

To investigate the effect of AS-IV on cell EMT, epithelial cell markers (E-cadherin and occludin) and mesenchymal cell markers (N-cadherin and vimentin) were measured using the Western blot analysis. As is shown in Figure 3A, E-cadherin and occludin were significantly down-regulated in the HG and HG with NC groups as compared with the control group. Further analysis revealed that E-cadherin and occludin expressions were significantly up-regulated in the AS-IV-treated groups as compared with the HG and HG with NC groups. However, the mesenchymal cell markers N-cadherin and vimentin demonstrated the opposite results in different groups (Figure 3A). In addition, these results were further confirmed using the immunofluorescence assay (Figure 3B,C). The E-cadherin intensity per cell was significantly decreased in the HG and HG with NC groups as compared with the control group, whereas the intensity increased in the AS-IV 40 or 80 μg/ml groups as compared with the HG and HG with NC groups (Figure 3D). However, the change in N-cadherin intensity per cell demonstrated opposing results in different groups (Figure 3E). These results suggest the inhibitory action of AS-IV on EMT in NRK-52E cells.

### The TGF-β1 activator inhibits NRK-52E cell viability and enhances cell apoptosis

**Figure 4:**
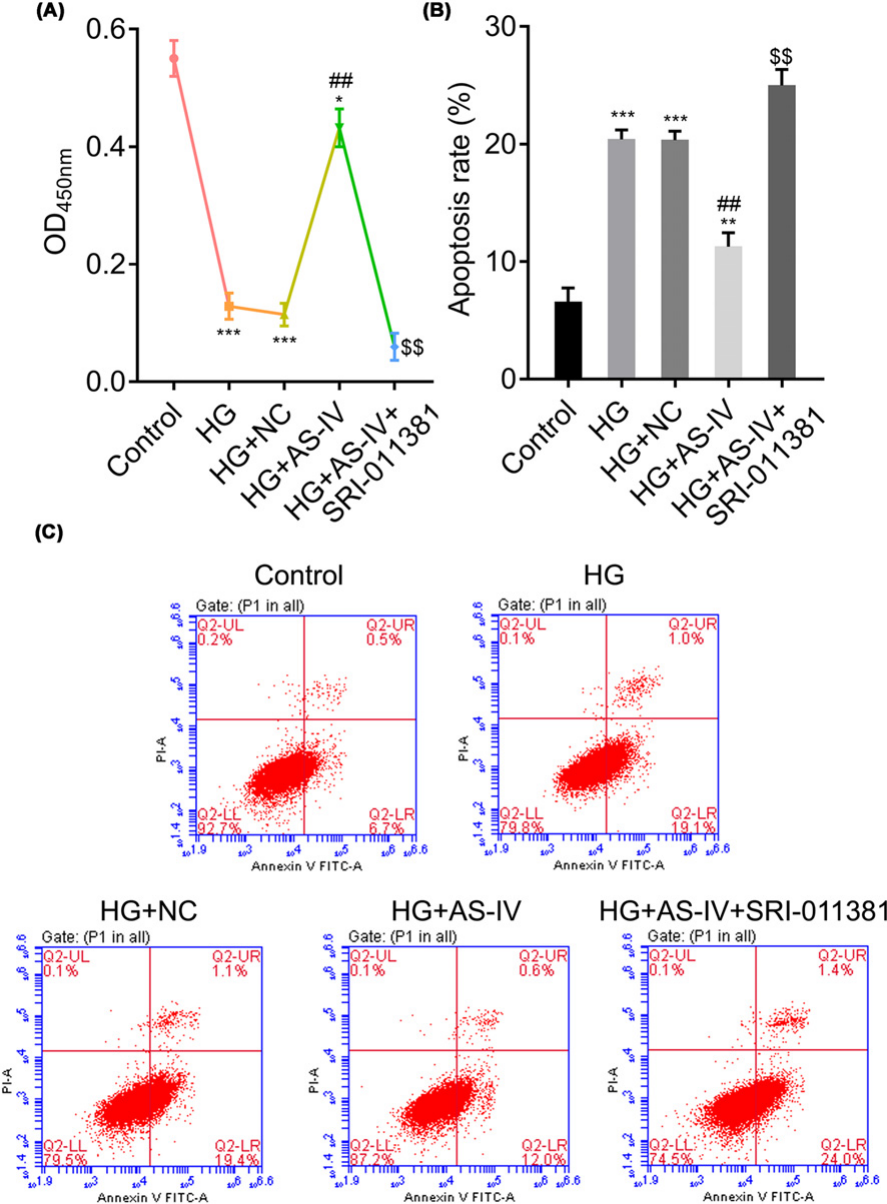
The TGF-β1 activator SRI-011381 modifies AS-IV-treated NRK-52E cell proliferation and apoptosis (**A**) Proliferation rates of NRK-52E cells. (**B,C**) The apoptosis rates of NRK-52E cells. Groups: HG; HG-induced NRK-52E cells; HG + NC, HG-induced NRK-52E cells with NC; HG + AS-IV, HG-induced NRK-52E cells with 80 μg/ml of AS-IV treatment. HG + AS-IV + SRI-011381, HG-induced NRK-52E treated with 80 μg/ml AS-IV, and the TGF-β1 activator SRI-011381. NRK-52E cells without any treatment served as the control group. All data are presented as mean ± SD. *P<0.05, **P<0.01, ***P<0.001 compared with the control group. ^##^*P*<0.01 compared with the HG group. ^$$^*P*<0.01 compared with the HG + AS-IV group.

To further examine and verify whether or not AS-IV participates in the TGF-β1-activated signaling pathway, the optimal concentration of AS-IV (80 μg/ml) and a TGF-β1 activator was selected for the next experiment. Overall, five groups were established: control, HG, HG with NC, HG with 80 μg/ml of AS-IV, and HG with AS-IV and SRI-011381 hydrochloride. First, we measured cell viability and apoptosis. As is shown in Figure 4, cell viability decreased significantly and cell apoptosis increased significantly in the HG with AS-IV and SRI-011381 hydrochloride group as compared with the HG and AS-IV group. These results were contrary to AS-IV; here, the TGF-β1 activator promoted cell apoptosis and inhibited cell viability and AS-IV conferred protective effects against SRI-011381 hydrochloride.

### The TGF-β1 activator up-regulates the TGF-β1 and Smad signaling pathway

**Figure 5:**
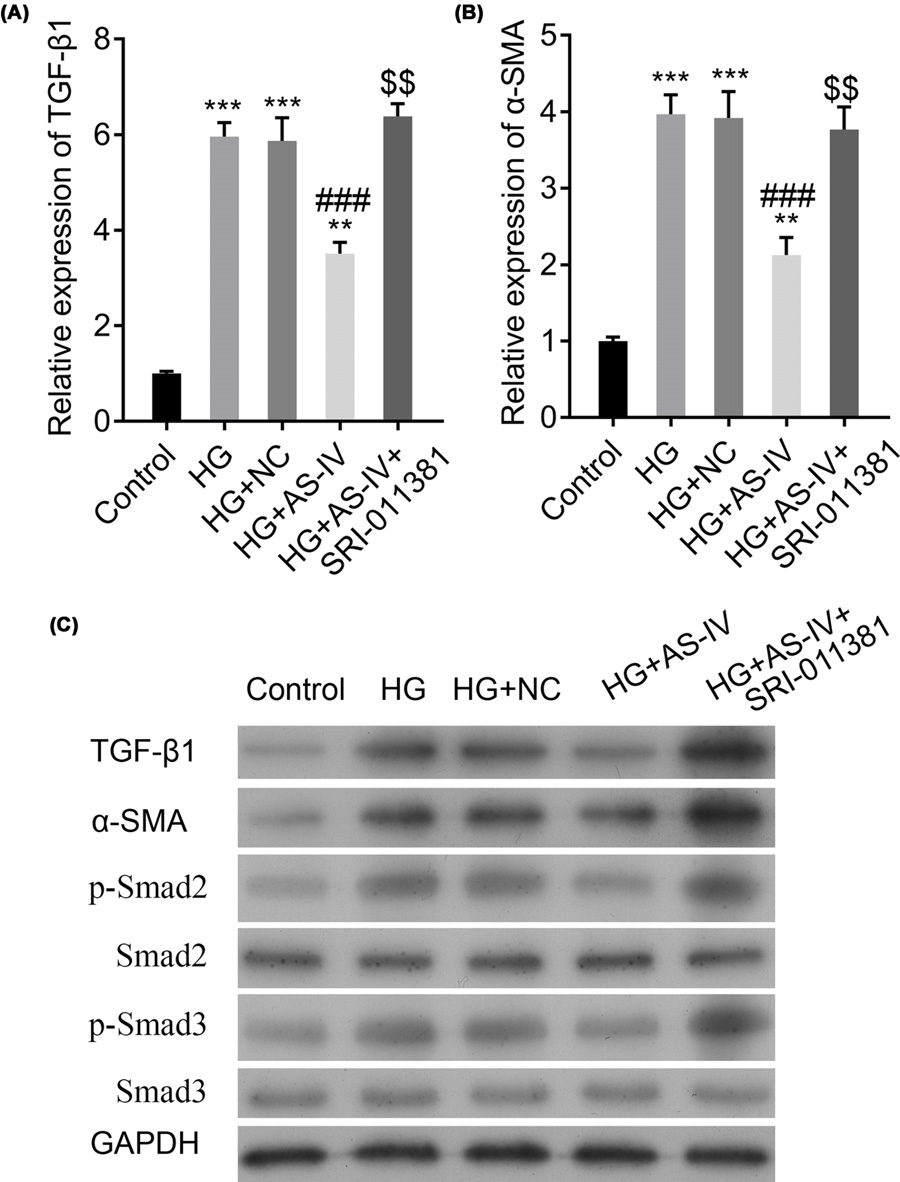
Changes in expressions of factors involved in the TGF-β1/Smad signaling pathway (**A,B**) The relative expressions of TGF-β1 and α-SMA, respectively, in different groups of NRK-52E cells. (**C**) The protein changes of factors involved in the TGF-β1/Smad signaling pathway. Groups: HG, HG-induced NRK-52E cells; HG + NC, HG-induced NRK-52E cells with NC; HG + AS-IV, HG-induced NRK-52E cells treated with 80 μg/ml of AS-IV. HG + AS-IV + SRI-011381, HG-induced NRK-52E cells treated with 80 μg/ml AS-IV, and the TGF-β1 activator SRI-011381. NRK-52E cells without any treatment served as the control group. All data are presented as mean ± SD. **P<0.01, ***P<0.001 compared with the control group. ^###^*P*<0.001 compared with the HG group. ^$$^*P*<0.01 compared with the HG + AS-IV group.

To elucidate whether the TGF-β1 activator up-regulates the TGF-β1 and Smad signaling pathway, we first measured the mRNA expressions of TGF-β1 and α-SMA. Contrary to the AS-IV-treated groups, the mRNA levels of TGF-β1 and α-SMA were significantly elevated in the HG with AS-IV and SRI-011381 hydrochloride group as compared with the HG and AS-IV group (Figure 5A,B). Consistent with the mRNA expression changes, the protein concentrations of TGF-β1 and α-SMA were significantly increased in the HG with AS-IV and SRI-011381 hydrochloride group as compared with the HG and AS-IV group. In addition, although the total protein concentrations of Smad2 and Smad3 remained unchanged, the phosphorylation level of Smad2 and Smad3 showed the same results with the changes of TGF-β1 and α-SMA in different groups by detecting the expression of p-Smad2 and p-Smad3 in protein level (Figure 5C). These findings suggest a possible modifying effect of the TGF-β1 activator on the TGF-β1 and α-SMA signaling pathway.

### The TGF-β1 activator modifies epithelial cell marker and mesenchymal cell marker proteins

**Figure 6:**
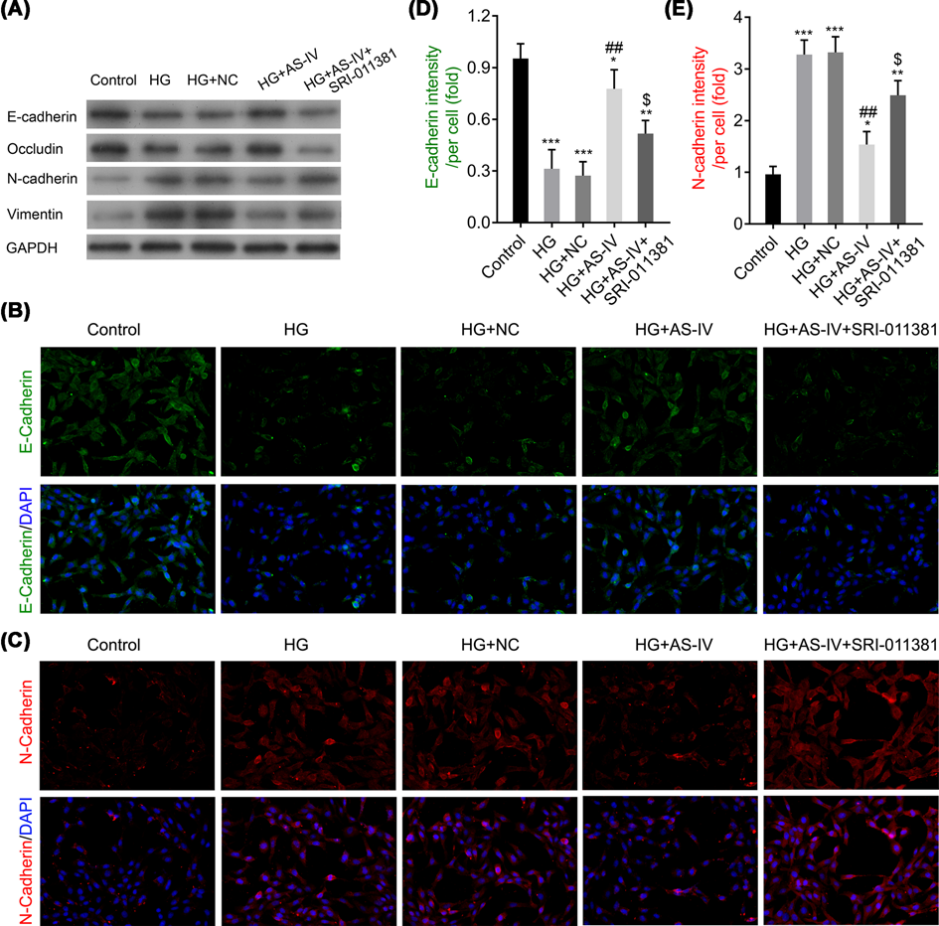
The effects of the TGF-β1 activator SRI-011381 on the expressions of E-cadherin, N-cadherin, vimentin, and occludin after AS-IV treatment (**A**) The protein expression levels of E-cadherin, N-cadherin, vimentin, and occludin. (**B,C**) The protein expression levels of E-cadherin and N-cadherin. (**D,E**) The intensity/per cell of E-cadherin and N-cadherin, respectively. Groups: HG, HG-induced NRK-52E cells; HG + NC, HG-induced NRK-52E cells with NC; HG + AS-IV, HG-induced NRK-52E cells treated with 80 μg/ml AS-IV; HG + AS-IV + SRI-011381, HG-induced NRK-52E cells treated with 80 μg/ml AS-IV, and the TGF-β1 activator SRI-011381. NRK-52E cells without any treatment served as the control group. All data are presented as mean ± SD. *P<0.05, **P<0.01, ***P<0.001 compared with the control group. ^##^*P*<0.01 compared with the HG group. ^$^*P*<0.05 compared with the HG + AS-IV group.

Meanwhile, we measured the marker proteins involved in cell EMT. The expressions of E-cadherin and occludin were significantly down-regulated in the HG with AS-IV and SRI-011381 hydrochloride group as compared with the HG with AS-IV group, whereas N-cadherin and vimentin demonstrated contrasting results between the HG with AS-IV and SRI-011381 hydrochloride group and the HG with AS-IV group (Figure 6A). These findings were further confirmed by the immunofluorescence assay (Figure 6B,C). The E-cadherin intensity per cell was significantly increased in the HG with AS-IV group as compared with the HG and the HG with NC groups, whereas the intensity decreased after treatment with the TGF-β1 activator (Figure 6D). However, the changes in N-cadherin intensity per cell demonstrated opposing results (Figure 6E).

## Discussion

DKD, a serious complication of diabetes, is the primary cause of ESRD worldwide. HG is associated with most functional and structural changes in renal PTCs [5,24]. Although accumulating evidence suggests an important role of AS-IV in EMT in renal PTCs [19,21], how it mediates its action remains unclear. In the present study, we examined the role of AS-IV in the HG-induced NRK-52E cell injury model. The results revealed the participation of AS-IV in TGF-β1 signaling, as was demonstrated by the rates of proliferation, apoptosis, and changes in TGF-β1 signaling-related protein expression, in NRK-52E cells subjected to AS-IV and TGF-β1 activator treatment.

Increased EMT significantly contributes to renal TIF, which is a hallmark pathological feature of DN. Previous study findings suggest EMT as a potential mechanism in HG-induced renal dysfunction that promotes renal fibrosis in rat PTCs [11,25,26]. Consistent with the previous study findings, our results revealed that AS-IV at concentrations ranging from 40 to 100 μg/ml could rescue viability and inhibit apoptosis in NRK-52 cells injured by HG treatment. In addition, EMT is characterized by phenotypic transition from epithelial cells to fibroblast-like cells. In this process, mesenchymal markers such as α-SMA, N-cadherin, and vimentin are induced and epithelial cell markers such as E-cadherin and occludin, which are essential for the structural integrity of the renal epithelium, are eliminated [17,27,28]. In the present study, HG increased α-SMA, N-cadherin, and vimentin expressions and reduced E-cadherin and occludin expressions, although contrary results were observed in the AS-IV-treated groups. These findings demonstrated that AS-IV could attenuate HG-induced EMT in renal PTCs.

EMT is a dynamic and complex process likely requiring the participation of growth factors or cytokines and the integration of multiple signal pathways at different stages. TGF-β1 is a major inflammatory factor in DN progression and a key mediator of EMT in PTCs. This action of TGF-β1 is principally facilitated via the activation of the MAPK, PI3K, Rho GTPase signaling pathway, and the Smad signaling pathway [29–33]. Lv et al. demonstrated that AS-IV could inhibit HG from inducing human tumor precursor cell (TPC) apoptosis by down-regulating TGF-β1 and inhibiting the p38MAPK pathway [9]. TGF-β1 phosphorylates Smad2 and Smad3 and subsequently associates with the common partner Smad4, which translocates into the nucleus and activates the expression of TGF-β-responsive genes [34]. Phosphorylated Smad2 and Smad3 were observed in the glomerular and mesenchymal areas of patients with diabetes, suggesting the important roles of Smad2 and Smad3 in EMT [35]. Few recent studies have reported that Smad3 silencing reduces the severity of renal fibrosis and that Smad2 promotes EMT by up-regulating CTGF and VEGF expressions in mice [25,36]. However, there are no studies on how AS-IV affects EMT in TPCs. To understand the possible protective mechanism of AS-IV on EMT, we analyzed the key molecules of the TGF-β1 and Smad signaling pathway, including TGF-β1, Smad2, Smad3, p-Smad2, and p-Smad3. We found that HG enhanced the expressions of these factors, whereas AS-IV reduced their expressions. To further elucidate the protective signaling mechanism of AS-IV on EMT, a TGF-β1 activator was added to AS-IV-treated NRK-52E cells. The results indicated that the TGF-β1 activator was able to exacerbate EMT, which was protected by AS-IV after HG treatment, suggesting the participation of AS-IV in the TGF-β1 and Smad signaling pathway. Overall, these findings suggested the protective role of AS-IV in TGF-β1/Smad-induced EMT.

## Conclusion

**Figure 7:**
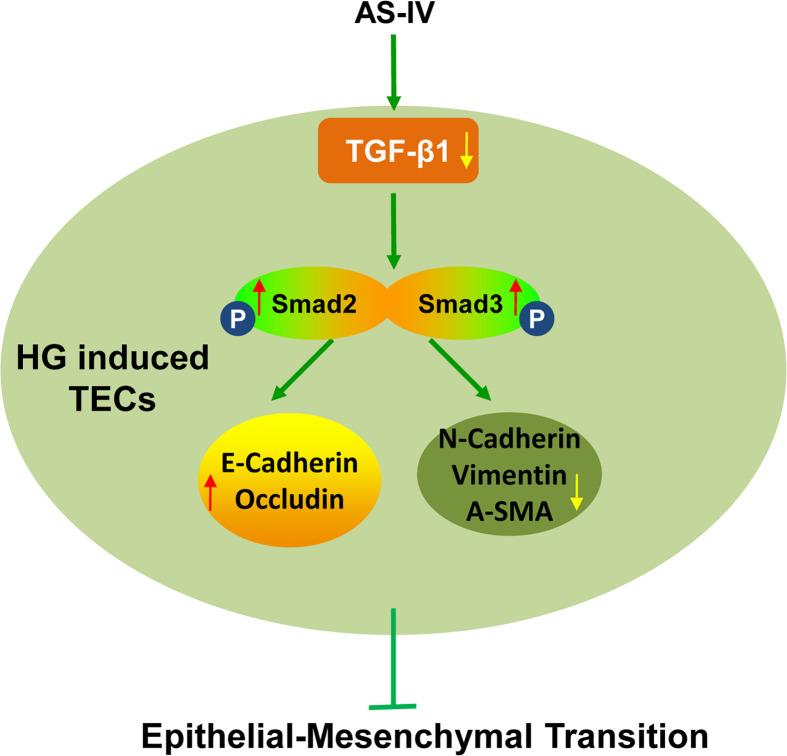
Schema of the regulatory mechanism of AS-IV on EMT of tubular epithelial cells AS-IV down-regulated TGF-β1 expression in HG-induced tubular epithelial cells (TECs), which promoted the phosphorylation levels of Smad2 and Smad3. The phosphorylation of Smad2 and Smad3 further promoted the expressions of the epithelial makers and inhibited the expressions of the mesenchymal markers, leading to EMT inhibition.

In conclusion, AS-IV is capable of attenuating HG-induced EMT by inhibiting the TGF-β1 and Smad pathway in renal PTCs (Figure 7). These findings could help establish novel therapeutic strategies to effectively prevent or arrest progressive renal failure.

## Abbreviations

AS-IV: astragaloside IV
CCK-8: cell counting kit-8
DKD: diabetic kidney disease
DN: diabetic nephropathy
EMT: epithelial-to-mesenchymal transition
ESRD: end-stage renal disease
FBS: fetal bovine serum
HG: high glucose
NC: negative control
PBS: phosphate buffered saline
PTC: proximal tubular epithelial cell
p-Smad2: phosphorylated-Smad2
qRT-PCR: quantitative real-time polymerase chain reaction
TGF-β: transforming growth factor-β
TIF: tubulointerstitial fibrosis
TPC: tumor precursor cell
α-SMA: α-smooth muscle actin
